# Diagnosis of Female Diverticula Using Magnetic Resonance Imaging

**DOI:** 10.1155/2008/213516

**Published:** 2008-06-23

**Authors:** Sima Porten, Stephanie Kielb

**Affiliations:** ^1^University of California San Francisco, San Francisco, CA 94143, USA; ^2^Department of Urology, Feinberg School of Medicine, Northwestern University, Chicago, IL 60611, USA

## Abstract

We investigate the ability of physical exam to diagnose urethral diverticula with or without magnetic resonance imaging (MRI) and exclusive of invasive modalities. A retrospective chart review of all women undergoing urethral diverticulectomy at our institution since 1999 was performed. We identified 28 female patients with a mean age at diagnosis of 42.6 years (range 18–66). Common presenting symptoms included dyspareunia, urgency, and frequency. Physical exam revealed a suspected urethral diverticulum in 26 (92.9%) patients, which was confirmed postoperatively in 17 of the 20 (85%) women who underwent surgical resection. Noninvasive imaging modalities (MRI or CT) were available for review in 20 (71%) cases and made the correct diagnosis of urethral diverticulum (presence or absence) in 19 (95%) patients. In those patients with symptoms of stress or urge incontinence (11, 39%), voiding cystourethrogram (VCUG) was performed. Urethral diverticula are often easily diagnosed on physical exam. MRI can be a useful adjunct for defining diverticular extent in surgical planning, especially for proximal and complex diverticula, and should be the modality of choice if clinical suspicion is high based on patient symptoms and physical exam.

## 1. INTRODUCTION

The incidence of urethral diverticula is thought to occur in 1–5% of the general female population [1, 2]. Presenting symptoms classically consist of dysuria, post void dribbling, dyspareunia, recurrent urinary tract infections (UTI), and stress incontinence. In fact, studies suggest that 1.4% of patients with stress incontinence have a urethral diverticulum [3]. Clinical diagnosis can be difficult due to the nonspecific nature of presenting symptoms and the possibility of concomitant genitourinary pathology.

However, it has been shown that in about 60% of cases a careful and thorough physical exam can make an accurate diagnosis of urethral diverticulum [3]. Ancillary modalities such as cystoscopy, voiding cystourethrogram (VCUG), and urethrography are reported to be diagnostic in 65–96% of cases, depending on the study [4–7]. Traditionally, the gold standard of diagnosis has been one or more of these ancillary and invasive techniques.

These invasive studies are difficult to perform properly and can be quite uncomfortable for patients. Recent advances and improvements in magnetic resonance imaging (MRI) have increased its use in the diagnosis of urethral pathology. MRI has multiplanar imaging capabilities with excellent tissue contrast, especially on T2-weighted images. Gadolinium enhancement can help define internal diverticular architecture [7]. Isolated studies in past literature demonstrated MRI to have a high sensitivity in the diagnosis of urethral diverticulum [7–10]. We report on a cohort of contemporary cases to determine whether the diagnosis of urethral diverticulum can be made on physical exam with or without MRI, exclusive of invasive cystoscopy or VCUG.

## 2. MATERIALS AND METHODS

Following IRB approval, female patients diagnosed with urethral diverticulum from 1999 to 2004 were identified by a retrospective chart review using electronic medical records and paper charts. Information about presenting symptoms, urological history, diagnosis method, imaging studies, and outcomes at last followup was documented. Surgical operative reports and pathology reports were also reviewed for final diagnosis.

Cases were reviewed to assess the method of initial diagnosis (physical exam, MRI, VCUG, cystoscopy, or urethrography). Our goal was to determine how the diverticula were diagnosed and to determine what studies were most sensitive at making the diagnosis. Therefore, any additional studies, as well as their contribution to diagnosis and surgical planning, were recorded. Outcomes at last followup were correlated with the type of imaging modality used for diagnosis and/or surgical planning.

MRI studies (if obtained) were performed at this institution using a 1.5 Tessla magnet with a phase-array pelvic coil. Axial, coronal, and sagittal T2 weighted sequences were obtained using fast spin echo technique. Axial and sagittal T1 weighted sequences were obtained before and after intravenous gadolinium contrast. Computer tomographic (CT) scans were used in four cases due to clinical contraindications for MRI.

## 3. RESULTS

We identified 28 female patients with a mean age at diagnosis of 42.6 years (range 18–66). The most common symptoms were dyspareunia in 13 (46%), urgency in 11 (39%), and frequency in 9 (32%) patients. Recurrent UTIs were common, occurring in 13 (46%) women; in one patient, it was the only symptom present. Of the patients with incontinence, 5 (18%) described post void dribbling characteristic for urethral diverticulum. Stress incontinence was present in 9 (32%) patients (Table 1). On physical exam, 26 (92.9%) patients were suspected to have a urethral diverticulum based on a visualized or palpable mass. Purulent material was expressed by compression in 3 (11.5%) masses. Of these patients, 20 (77%) underwent surgical resection, with the remaining 6 patients either found to have no abnormalities on further imaging or were essentially lost to follow up. A postoperative diagnosis of urethral diverticulum was correctly made in 17 (85%) patients and vaginal wall cyst was the final diagnosis in 3 patients. Two patients who did not have physical exam findings underwent surgical resection based on MRI findings. One was found to have a diverticulum, and the other had no pathology found at time of surgical exploration (Table 2).

Noninvasive imaging modalities (MRI or CT) were available for 20 (71%) of the 28 cases reviewed and 19 (95%) of these patients underwent surgical excision. MRI and CT made the correct diagnosis in 16 (100%) cases with postoperative diagnosis of urethral diverticulum. Three patients did not have a final diagnosis of urethral diverticulum. Of these, MRI and CT correctly stated that 2 (67%) were not urethral diverticulum, and incorrectly stated that 1 (33%) was a urethral diverticulum. The one patient who did not undergo surgery was diagnosed with urethral trauma based on MRI findings. In summary, the correct diagnosis of urethral diverticulum (presence or absence) was made in 19 (95%) patients (Table 3).

**Figure 1:**
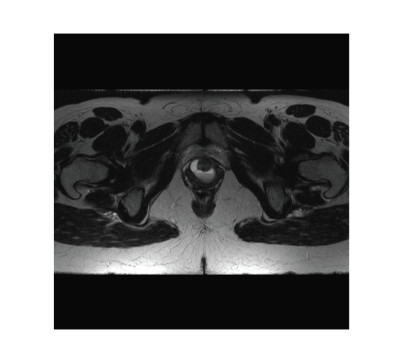
Urethral diverticulum on MRI (T2 weighted, fast spin echo). Patient presented with dyspareunia, urinary urgency and a normal physical exam. VCUG showed no evidence of urethral diverticulum. MRI revealed the correct diagnosis.

In our cohort, videourodynamics (VCUG) was performed in 11 (39%) patients with symptoms of stress or urge incontinence. Those with documented Type II stress incontinence (5 patients) also underwent a pubovaginal sling procedure at time of diverticular resection. No patient had a diverticulum detected exclusively on VCUG that had not previously been diagnosed on physical exam. Of note, one patient had a proximal diverticulum diagnosed by MRI alone, which was not picked up on physical exam or on VCUG (Figure 1). A total of 8 (30.8%) out of 28 patients had no imaging studies (based on clinical contraindications like pregnancy), or were lost to follow up (Table 4).

MRI and CT showed diverticula at an average of 11.1 cm^3^ (range 0.65–69.8 cm^3^). The most common histological finding described by pathology was “chronic inflammation” in 9 (47%) of 19 patients with surgical pathology available for review. Nephrogenic adenoma was found in one specimen and in no patient was diverticular carcinoma found.

The average time at the last followup was 11.9 months (ranging from 0.5–48 months). No patient (with or without preoperative VCUG) developed de novo urinary incontinence by history, though postoperative urodynamics testing was not routinely employed. A single patient had a recurrent urethral diverticula confirmed by MRI and underwent repeat excision for recurrence 1 year after initial resection. This patient had a large and complex diverticulum with significant proximal extension.

## 4. DISCUSSION

A high index of suspicion is necessary to initiate the appropriate steps for diagnosis of urethral diverticula. Based on past studies and reaffirmed in our cohort, once suspected, a good physical exam can easily and reliably hint at the correct diagnosis; 17 out of 20 (85%) women were eventually found to have urethral diverticulum based on suspicious physical exam findings. MRI was diagnostic in one patient in whom physical exam findings were lacking and who had a proximal diverticulum, and a useful adjunct in defining diverticular anatomy for surgical planning. As no patient had a diverticula diagnosed with adjunctive modalities, these are not needed and should not be a routine component of patient evaluation. Our opinion is that videourodynamics should be considered only in those patients with concomitant stress incontinence symptoms or evidence of other genitourinary pathology.

Studies from the early 90’s showed that MRI is sensitive and specific for the detection of urethral diverticulum, but its high cost and relative unavailability prohibited its use as a diagnostic tool [11]. Since that time, improvements in MRI technology and availability have increased its use in the diagnosis of urethral diverticulum. Daneshgari et al. [8] reported three cases of intraurethral wall diverticula diagnosed by MRI after cystoscopy, VCUG, and ultrasonography had failed. Kim et al. examined 20 cases of urethral diverticula verified surgically, showing that MRI had a sensitivity of 70%, as compared with urethrography (VCUG and double balloon) or urethroscopy, each with sensitivity of 55%. Despite these findings, urethroscopy followed by VCUG was recommended for initial evaluation due to the high cost of MRI at the time of the study. Five years later, Neitlich et al. [9] reported that MRI could detect urethral diverticula in women who had negative double-balloon urethrography and VCUG studies. A T2 weighted, fast spin echo technique was used with a dedicated pelvic multicoil, creating a faster and more cost-effective technique as compared to Kim et al. To date, there has been no study directly comparing VCUG with surface MRI, although Blander et al. [10] retrospectively demonstrated that endoluminal MRI was more accurate than VCUG in determining size and extent of urethral diverticula in 27 women.

In contrast to these studies, A. C. Wang and C. R. Wang [4] supported the use of VCUG and positive pressure urethrogram in the diagnosis of urethral diverticula due to their high combined sensitivity of 100%. In their study, MRI is noted as a better modality overall, but is discounted as a primary imaging technique due to its high cost. In general, the recent literature supports the superiority of MRI over invasive imaging modalities by proving its sensitivity and specificity in detecting urethral pathology, through valid prospective and retrospective trials [7–10]. Cost effectiveness is also possible with newer MRI techniques and increased availability or access to MRI machines, although it is still somewhat of an issue. In addition, multiple case reports comment on the value of MRI in elucidating confusing, atypical, or complex presentations. Our contemporary chart review supports and extends the growing consensus that MRI is the new gold standard in diverticular diagnosis, as noninvasive imaging modalities made the correct diagnosis of presence or absence of a urethral diverticulum in 19 out of 20 (95%) patients.

Although VCUG has been shown to be effective in detecting urethral diverticula, inadequate distention of the urethra or stenotic diverticular ostia can decrease its sensitivity as compared to other invasive studies [4]. In our cohort, VCUG added little to the accurate diagnosis and treatment of a urethral diverticulum. We showed that eliminating this invasive study does not impact on operative outcomes or diverticular recurrence. However, videourodynamics may still have a role in the evaluation of patients with urethral diverticula, due to the high association of concomitant stress incontinence. VCUG would be a useful study, when surgical treatment for incontinence is desired by the patient, in addition to diverticular resection [12]. Therefore, both procedures can be performed at once saving the patient an additional operating room visit. VCUG may also be useful to evaluate for other genitourinary pathology such as obstruction (if symptoms warrant) and to help define preoperative voiding dysfunction as mentioned above. Otherwise, VCUG is not a necessary procedure for the routine evaluation of urethral diverticula.

It is also unclear if patients with a diverticulum diagnosed on physical exam need to undergo any imaging study. We still consider MRI a useful adjunct and advocate its use, especially in defining the proximal extension of complex diverticula, as seen in our patient who was diagnosed with MRI alone (Figure 1). Currently, it is thought that patients with significant proximal extension are at an increased risk for postoperative urinary incontinence. In these cases, high-resolution imaging may play an important role in preoperative counseling as well as surgical planning. However with a small, distal, obvious diverticulum seen on physical examination (in the absence of stress incontinence), proceeding directly to operative excision is a reasonable, cost-effective approach if MRI is not available.

There are some limitations to our study that deserve mention. As with any retrospective chart review, there is reviewer dependent bias. Although our study was relatively large compared to other studies in the literature, our sample size is still somewhat small. Since charts were pulled based on diagnosis codes, different physicians performed the physical exam and VCUG, which creates a chance for variability based on individual skill and experience. We are a tertiary care centre, therefore a few of these patients may have come with a preexisting diagnosis. This likely produces only a small amount of bias since a large part of our practice is general urology with many referrals from primary care physicians within our hospital system. Only three patients in our cohort were referred from community urologists with a preexisting diagnosis. Different radiologists read the MRI findings, also creating interoperator variability. The mean size of diverticula seen on imaging was large (11.1 cm^3^), which could play a role in the sensitivity of physical exam. In addition, not all patients underwent both VCUG and MRI evaluation, so no direct comparison of imaging modalities is possible. For future studies, a prospective trial comparing MRI directly with VCUG is needed to definitively establish which modality has a superior sensitivity and specificity in the diagnosis and management of urethral diverticula, as well as overall cost effectiveness.

## 5. CONCLUSION

Urethral diverticula can be easily diagnosed on physical exam based on symptoms and clinical suspicion. MRI can be a useful adjunct for defining diverticular extent in surgical planning especially in women with proximal diverticula; it should be the modality of choice if patient presentation is suggestive and physical exam findings are lacking. Additional invasive imaging, like VCUG, may still have a role in patients with concomitant urological disease. Otherwise, VCUG adds little to accurate diagnosis and treatment of urethral diverticula, and its elimination does not impact accurate diagnosis or operative outcomes.

## Figures and Tables

**Table 1:** Patient characteristics.

Mean age (range)	42.6 (18–66)
No. symptoms (%)
Dyspareunia	13 (46)
Urgency	11 (39)
Frequency	9 (32)
Dysuria	8 (29)
Recurrent urinary tract infection	13 (46)
Stress incontinence	9 (32)
Post void dribbling	5 (18)

**Table 2:** Physical exam (PE) findings in 24 patients with definitive diagnosis. Definitive diagnosis was made by either surgery or imaging studies.

	Final diagnosis of urethral diverticulum		
Suspected diverticulum on PE	Yes	No	

Yes	17	3	20
No	1	1	2

	18	4	

** **Sensitivity (%): 17/18 (94) Specificity (%): 1/4 (25) Positive predictive value (%): 17/20 (85) negative predictive value (%): 1/2 (50).

**Table 3:** Noninvasive imaging findings in 20 patients.

No	0	3	3
	16	4	

** **Sensitivity (%): 16/16 (100) Specificity (%): 3/4 (75) Positive predictive value (%): 16/17 (94) negative predictive value (%): 3/3 (100).

**Table 4:** VCUG in patients with symptoms of incontinence.

No. of patients with VCUG (%)	11 (55)
Type II stress incontinence	5 (45)
VCUG (+) diverticula	5 (45)
MRI/CT (+) diverticula	5 (45)
MRI/CT (−) diverticula	0 (0)
VCUG (−) diverticula	6 (55)
MRI/CT (+) diverticula	6 (55)
MRI/CT (−) diverticula	0 (0)
